# Geographic Correlation between Tapeworm Carriers and Heavily Infected Cysticercotic Pigs

**DOI:** 10.1371/journal.pntd.0001953

**Published:** 2012-12-20

**Authors:** Seth E. O'Neal, Luz M. Moyano, Viterbo Ayvar, Guillermo Gonzalvez, Andre Diaz, Silvia Rodriguez, Patricia P. Wilkins, Victor C. W. Tsang, Robert H. Gilman, Hector H. Garcia, Armando E. Gonzalez, The Cysticercosis Working Group in Peru

**Affiliations:** 1 Department of Public Health and Preventive Medicine, Oregon Health and Science University, Portland, Oregon, United States of America; 2 Cysticercosis Elimination Program and Center for Global Heath Tumbes, Universidad Peruana Cayetano Heredia, Tumbes, Peru; 3 Instituto de Ciencias Neurológicas, Lima, Peru; 4 Centers for Disease Control and Prevention, Atlanta, Georgia, United States of America; 5 Georgia State University, Atlanta, Georgia, United States of America; 6 Department of Microbiology, School of Sciences, Universidad Peruana Cayetano Heredia, Lima, Peru; 7 Department of International Health, Bloomberg School of Public Health, Johns Hopkins University, Baltimore, Maryland, United States of America; 8 School of Veterinary Medicine, Universidad Nacional Mayor de San Marcos, Lima, Peru; Universidad Nacional Autónoma de México, Mexico

## Abstract

**Background:**

Neurocysticercosis is a leading cause of preventable epilepsy in the developing world. Sustainable community-based interventions are urgently needed to control transmission of the causative parasite, Taenia solium. We examined the geospatial relationship between live pigs with visible cysticercotic cysts on their tongues and humans with adult intestinal tapeworm infection (taeniasis) in a rural village in northern Peru. The objective was to determine whether tongue-positive pigs could indicate high-risk geographic foci for taeniasis to guide targeted screening efforts. This approach could offer significant benefit compared to mass intervention.

**Methods:**

We recorded geographic coordinates of all village houses, collected stool samples from all consenting villagers, and collected blood and examined tongues of all village pigs. Stool samples were processed by enzyme-linked immunosorbent assay (ELISA) for presence of Taenia sp. coproantigens indicative of active taeniasis; serum was processed by enzyme-linked immunoelectrotransfer blot for antibodies against T. solium cysticercosis (EITB LLGP) and T. solium taeniasis (EITB rES33).

**Findings:**

Of 548 pigs, 256 (46.7%) were positive for antibodies against cysticercosis on EITB LLGP. Of 402 fecal samples, 6 (1.5%) were positive for the presence of Taenia sp. coproantigens. The proportion of coproantigen-positive individuals differed significantly between residents living within 100-meters of a tongue-positive pig (4/79, 5.1%) and residents living >100 meters from a tongue-positive pig (2/323, 0.6%) (p = 0.02). The prevalence of taeniasis was >8 times higher among residents living within 100 meters of a tongue-positive pig compared to residents living outside this range (adjusted PR 8.1, 95% CI 1.4–47.0).

**Conclusions:**

Tongue-positive pigs in endemic communities can indicate geospatial foci in which the risk for taeniasis is increased. Targeted screening or presumptive treatment for taeniasis within these high-risk foci may be an effective and practical control intervention for rural endemic areas.

## Author Summary

*Taenia solium*, aka the pork tapeworm, is an important cause of epilepsy in developing nations. People with intestinal tapeworms, a condition known as taeniasis, pass infectious eggs in their feces which contaminate the environment. These eggs can cause serious disease in both humans and pigs if they are ingested. Treating taeniasis is one way to potentially control transmission of the parasite in affected communities. However, this is difficult because people with taeniasis usually have no symptoms and therefore don't know they are infected. As a result, control programs may resort to offering treatment to entire communities in order to reach a few tapeworm carriers. Focusing detection and treatment on high-risk subgroups is another approach which might reduce unnecessary treatments. In this study, we found that people with taeniasis are more likely to be found living nearby pigs with visible signs of infection, specifically tapeworm cysts on their tongues. This suggests that routine tongue examination by pig owners and buyers could identify neighborhoods where detection and treatment of taeniasis may be more efficient.

## Introduction

*Taenia solium*, otherwise known as the pork tapeworm, is a common helminthic infection of the human central nervous system (CNS) and a leading cause of acquired epilepsy in low and middle income countries. Neurocysticercosis (NCC) is a disease which occurs when *T. solium* larval cysts infect the CNS causing a broad range of neurologic manifestations, including seizures, headache, intracranial hypertension, hydrocephalus, encephalitis, stroke, cognitive impairment, and psychiatric disturbances [1], [2]. In endemic areas, *T. solium* infection is a major cause of epilepsy with 30% of seizure disorder attributable to NCC [3]–[5]. In Latin America alone an estimated 400,000–1.35 million people have seizure disorders secondary to NCC [6], [7]. Effective resource-appropriate control and elimination strategies are urgently needed to prevent additional disease.

Humans are the definitive host of the adult intestinal tapeworm, a condition known as taeniasis. People with taeniasis shed tapeworm eggs in their feces which contaminate the environment, particularly in rural regions where open defecation is common. When pigs are allowed to roam and consume human feces they are at risk of contamination with *T. solium* eggs and infection with intermediate stage larval cysts in their tissues (cysticercosis). The parasite lifecycle completes when humans consume these larval cysts in undercooked pork, which in turn develop into mature adult tapeworms in the intestine capable of shedding infective eggs. Humans acquire cysticercosis including NCC through incidental ingestion of *T. solium* eggs in fecal contamination.

Treatment of taeniasis is a key component of control and elimination strategies as adult intestinal tapeworms are the immediate common source of cysticercosis in both human and pigs. However, direct identification of taeniasis is complicated by low prevalence in endemic communities and by asymptomatic clinical course of infection [8]. Mass treatment with either niclosamide or praziquantel has been applied in several settings with modest effect [9]–[12]. These drugs are available in single-dose oral regimens and are reported to be 90–95% efficacious for eliminating taeniasis [13]. However, control gains are temporary without repeated interventions [12]. An alternative strategy is to focus resources in specific sub-populations in which the prevalence of taeniasis is increased [14]. Targeting high-risk foci can have substantial benefits in terms of the number of treatments administered, the frequency of adverse events related to treatment and the overall prevalence of infection in the community [15]. However, practical methods to identify high-risk foci of taeniasis are needed in order to apply this approach.

It is biologically plausible that pigs infected with a heavy-burden of *T. solium* cysts could serve as indicators for high-risk geographic foci of taeniasis within endemic villages. These heavy-burden pigs presumably have increased or repeated exposure to feces contaminated with *T. solium* eggs, which suggests geographic proximity to a case of taeniasis. Exposure was also presumably relatively recent as most pigs raised for consumption have a short lifespan. Pigs with heavy-cyst burden can be identified by examination of the tongue or by visual inspection of the meat at the time of slaughter. Villagers in many parts of Latin America are already familiar with the tongue-exam, as this method of inspection is commonly practiced at the time a pig is sold. From a control perspective, this method of identifying high-risk foci of taeniasis has operational advantages in that surveillance can therefore be community-based. Screening or presumptive treatment for taeniasis can then be targeted to within geographic rings centered on the house where the index pig was raised. The objective of this study was to examine the geospatial relationship between live pigs with visible cysts in their tongues and human taeniasis in an endemic community.

## Methods

### Hypothesis

The hypothesis being tested is that the prevalence of taeniasis is higher among households in the immediate vicinity of a tongue-positive pig compared to households that are distant from the tongue-positive pig.

### Study Site and Participants

The study was conducted in the rural village of Rica Playa, in the Department of Tumbes, Peru. The northern coastal region of Peru is known to be endemic for *T. solium* with high rates of transmission of cysticercosis to both humans and pigs and substantial neurologic disease attributed to NCC [5], [16]. Agriculture is the main economic activity in the region and villagers frequently raise pigs both for consumption and sale. Pigs are typically unrestrained and are allowed to roam freely to forage for food. This practice reduces the owner's overall cash investment in feed but potentially exposes their pigs to *T. solium* eggs in fecal contamination as latrines are limited and open defecation is common. All village residents 2 years of age and older were eligible to participate in this study. Children younger than 2 years old were excluded as taeniasis is very uncommon in this age group.

### Field Procedures

We conducted an initial census by visiting each household in the community and recording the age and sex of all resident household members, the number of pigs raised in the household, and the source of water and type of sanitary facilities available. We considered any individual who slept more than 2 nights per week in the village to be a resident. Latitude and longitude coordinates of each house were recorded using hand-held global positioning system (GPS) receivers (GeoExplorer II; Trimble, Sunnyvale, CA) with post-processed differential correction for sub-meter accuracy.

We then distributed a 500-ml plastic container with lid to all consenting residents ≥2 years old for collection of a whole stool sample. We also collected a 5-ml peripheral blood sample via venipuncture in standard serology vacuum tubes. Blood samples were maintained in coolers with ice-packs while in the field. All blood and stool samples were transported daily to the laboratory facility in Tumbes for processing.

Field teams captured all household pigs and a veterinarian inspected the tongue for nodules characteristic of *T. solium* cysticercosis. To examine the tongue, the animal was manually restrained and a hard wooden pallet was used to keep the mouth open. A veterinarian then gently retracted the tongue with a cloth visually inspecting and palpating the entire length of the underside of the tongue. The pig was considered positive for cysticercosis if cyst-like nodules were either seen or felt [17]. We collected a 5-ml blood sample from the vena cava of all pigs and transported these in the same manner as the human samples.

### Laboratory Procedures

All samples were first processed in the laboratory facilities of the Global Health Center in Tumbes, Peru. Whole stool samples were examined macroscopically for the presence of *Taenia sp*. scoleces or proglottids. A 10cc fecal sample was then placed in 40 cc of 5% formol-Phosphate Buffered Saline, pH 7.2 (PBS) in a sealed propylene tube at room temperature. Blood samples were centrifuged and aliquoted in 1.5 ml microtubules at −20°C. These serum and fecal samples were then shipped by air to the CNS Parasitic Diseases Research Unit, Universidad Peruana Cayetano Heredia (Lima, Peru) for further analysis.

Fecal samples were concentrated by sedimentation then examined by light microscopy for the presence of *Taenia sp*. eggs or proglottids. Fecal samples were also processed with an enzyme-linked immunosorbent assay (ELISA) to detect *Taenia sp.* coproantigens as previously described [18]. However, we used a more conservative cutoff to increase the overall specificity for detection of *Taenia sp*. and to decrease the likelihood of detection of *T. saginata* taeniasis, a co-endemic cestode species. For each sample a percent positivity (PP) value was calculated as the optical density (OD) value of the sample relative to the OD of the positive control. A PP≥14% was considered positive.

Human and pig sera samples were analyzed by enzyme-linked immunoelectrotransfer blot for presence of antibodies against *T. solium* cysts (EITB LLGP) as previously described [19]. The EITB LLGP assay uses a semi-purified fraction of homogenized *T. solium* cysts containing 7 *T. solium* glycoprotein antigens named after the Kda molecular weights of the corresponding reactive bands (GP50, GP42, GP24, GP21, GP18, GP14, GP13). Reaction to any of these 7 glycoprotein antigens is considered positive. Reaction to 4 or more bands is associated with heavy infection and viable cysts in pigs [20]. This assay is considered to be 100% specific to the *T. solium* metacestode stage with no cross-reactions with other co-endemic *Taenia sp.* Human sera were also analyzed by EITB for presence of antibodies against recombinant antigens specific to *T. solium* adult stage infection (EITB rES33). The EITB rES33 is based on baculovirus expression-purified recombinant antigen rES33 [21].

### Ethics

This study was reviewed and approved by the Institutional Review Board and the Institutional Ethics Committee for the Use of Animals at the Universidad Peruana Cayetano Heredia, Lima, Peru. All participants provided written informed consent, with parental or guardian consent required for the participation of minors. Treatment of animals adhered to the Council for International Organizations of Medical Sciences (CIOMS) International Guiding Principles for Biomedical Research Involving Animals.

### Statistical Methods

We entered individual household coordinates into ArcMAP10 GIS software (ESRI; Redlands, CA) to generate a geo-referenced map of the community which included results of tongue-examination and laboratory exams. We used these coordinates to calculate the geodesic distance in meters from each household to the nearest house where a tongue-positive pig was raised.

All data were analyzed in STATA SE12 (StataCorp; College Station, TX). Fisher's exact test was used to compare distributions of proportions or to examine association between pairs of categoric measures. A score test was used to evaluate linear trend in log odds across categories. We constructed univariate logistic regression models with random-effects to estimate the odds of a positive test result while accounting for clustering at the household level. All tests were 2-sided, and significance was set at 0.05. We then used binomial family generalized estimating equations (GEE) with a log link and robust sandwich-type standard errors to estimate the population-averaged proportions of positive test results among household residents 1) living 100 meters or less from a house where a tongue-positive pig was found (includes index household) and 2) living more than 100 meters from a house where a tongue-positive pig was found. The 100-meter distance was chosen as a familiar measurement which could be readily applied in a community-based control intervention. We used households as the clustering variable and applied Quasilikelihood Information Criteria (QIC) in a STATA module to determine which variables to include [22], [23]. Only those variables which decreased the QIC value compared to that of a full model were included in the final GEE algorithm.

## Results

### Human Population and Sampling

There were a total of 454 residents living in the study area at the time of the census including 240 (52.9%) males and 214 females (47.1%). The median age was 28 years (interquartile range [IQR] 14–47) with an overall range of 0–95 years. Residents were distributed among 101 different households (Table 1).

**Table 1:** Characteristics of households, Rica Playa, Tumbes, Peru.

Household characteristics, n = 101	
Average no. of houses per 100 meters radius	7.8
No. residents per household, median [IQR]	4 [2]–[6]
No. rooms per household, median [IQR]	5 [4]–[6]
No. households with latrines, (%)	46 (47.4)
No. households raising pigs, (%)	73 (80.2)
No. pigs per household, median [IQR]	4 [2]–[9]
No. households with ≥1 seropositive pig with more than 1 reactive band on EITB LLGP, (%)	62 (61.4)
No. households with ≥1 seropositive pig with 4–7 reactive bands on EITB LLGP, (%)	8 (8.0)
No. households with ≥1 tongue-positive pig, (%)	7 (6.9)
Distance in meters to nearest home with tongue-positive pig, median [IQR]	448 [137–905]

IQR = interquartile range.

Blood samples were obtained from 385 residents aged 2 years or older for a total blood sampling coverage of 84.8%. All 385 individuals who provided blood also provided a stool sample, and an additional 17 (3.7%) provided stool but no blood. A total of 402 residents aged 2 years or older provided a stool sample for 88.5% coverage of the total population. There were 52 (11.5%) unsampled individuals who provided neither blood nor stool, including 12 children <2 years old. People who did not provide samples were more likely to be males and also to raise pigs (data not shown). There were 47(10.4%) residents in 10 households who provided stool or blood samples but did not allow their pigs to be tested.

### Swine Population and Porcine Cysticercosis

We captured 548 pigs in the village, of which 256 (46.7%) were positive for antibodies against one or more bands on EITB LLGP for cysticercosis (Table 2). The positive band distribution on EITB LLGP was as follows: 109 (42.6%) with a single band, 139 (54.3%) with 2–3 bands and 8 (3.1%) with 4–7 bands. In all 109 single-band positive samples the positive band corresponded to the 50 Kd glycoprotein (gp50). There were 11 pigs (2.0%) that were positive for cysticercosis on tongue exam. Of these 11 tongue-positive pigs, 10 (90.9%) were seropositive on EITB LLGP.

**Table 2:** Relationship between household characteristics and *Taenia solium* cysticercosis in pigs, Rica Playa, Tumbes, Peru.

	EITB LLGP (1 or more reactive bands)		EITB LLGP (4–7 reactive bands)		Tongue exam	
Variable	No. positive, (%)	p*	No. positive, (%)	p*	No. positive, (%)	p*
No. residents per household						
1–5 (1^st^ tertile)	147 (44.0)	0.29	6 (1.8)	0.89	5 (1.5)	0.29
6–7 (2^nd^ tertile)	50 (51.0)		1 (1.0)		4 (4.1)	
8–10 (3^rd^ tertile)	59 (50.9)		1 (0.9)		2 (1.7)	
No. rooms per household						
1–4 (1^st^ tertile)	87 (41.8)	0.07	4 (1.9)	0.90	5 (2.4)	0.92
5–6 (2^nd^ tertile)	112 (47.1)		3 (1.3)		4 (1.7)	
7–9 (3^rd^ tertile)	57 (55.9)		1 (1.0)		2 (2.0)	
No. houses per 100 meters						
1–5 (1^st^ tertile)	118 (47.4)	<0.01	3 (1.2)	0.30	4 (1.6)	<0.01
6–9 (2^nd^ tertile)	85 (60.7)		4 (2.9)		7 (5.0)	
10–19 (3^rd^ tertile)	53 (33.3)		1 (0.6)		0 (0)	
Latrine in household						
Yes	97 (46.4)	0.93	5 (1.6)	1.0	5 (2.4)	0.76
No	159 (46.9)		3 (1.4)		6 (1.9)	
No. pigs per household						
1–3 (1^st^ tertile)	30 (55.6)	0.37	3 (5.6)	0.04	0 (0)	0.52
4–8 (2^nd^ tertile)	74 (46.8)		2 (1.3)		2 (1.3)	
9–26 (3^rd^ terile)	152 (45.2)		3 (0.9)		9 (2.7)	

* Fisher's exact test, two-sided.

### Human Taeniasis

**Figure 1:**
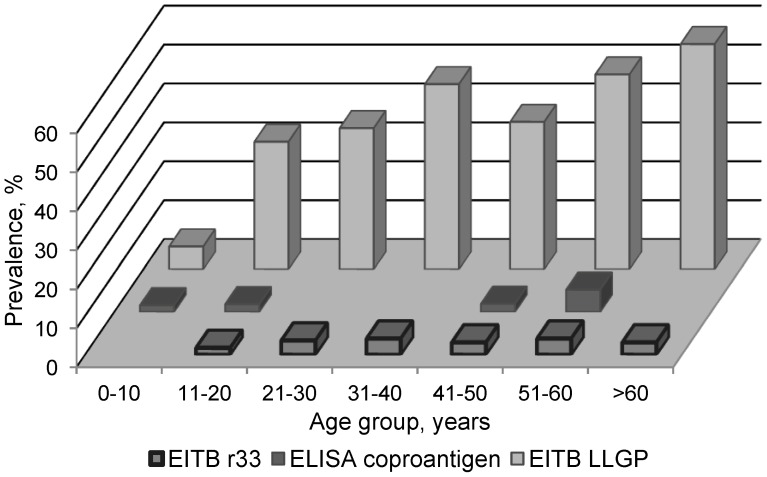
Relationship between age category and positive laboratory results for *Taenia solium* infection in humans, Rica Playa, Tumbes, Peru.

**Figure 2:**
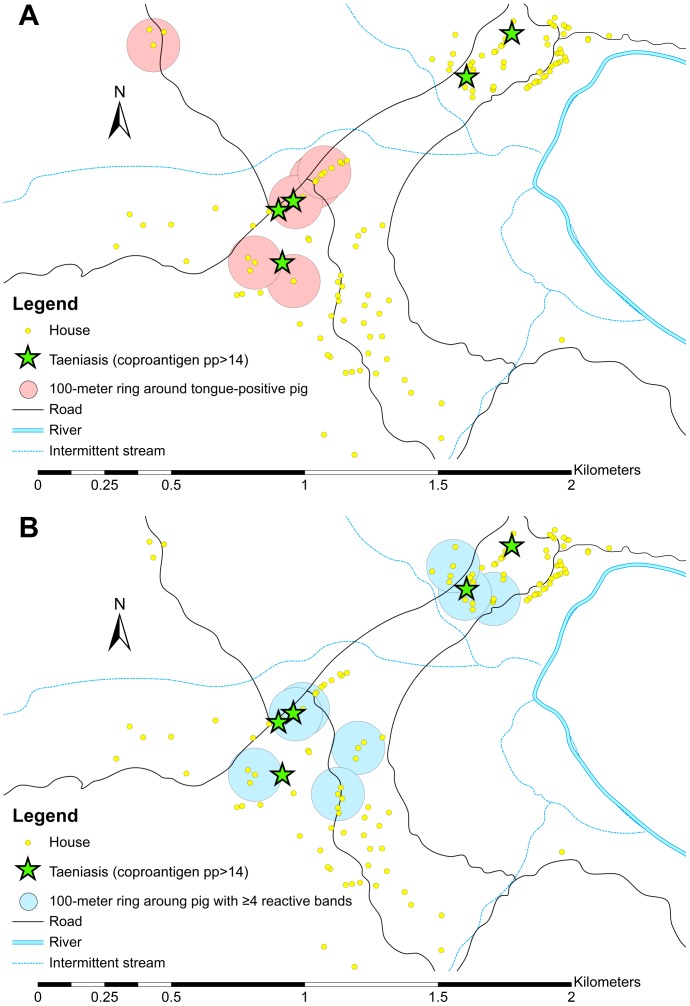
Maps of Rica Playa, Tumbes, Peru, showing the geographic locations of people with taeniasis and heavily-infected cysticercotic pigs. Green stars indicate the 5 households in which 6 coproantigen-positive individuals resided. A. Red circles indicate 100-meter ring radius around households where a tongue-positive pig was raised. There were 11 tongue-positive pigs raised in 7 different households (2 overlap on map). B. Blue circles indicate 100-meter ring radius around households where a seropositive pig with more than 4 reactive bands on EITB LLGP was raised. There were 8 pigs with 4+ reactive bands in 8 separate households.

Of the 402 fecal samples, 6 (1.5%) were positive for the presence of *Taenia sp*. coproantigens. *Taenia sp*. eggs were seen by light microscopy in only 1 (16.7%) of these 6 samples. No other samples had *Taenia sp*. eggs present. There was no statistical difference between coproantigen-positive residents and coproantigen-negative residents with respect to age (Figure 1), sex or most demographic characteristics with the exception of household distance to a tongue-positive pig (Table 3). Of the 6 total coproantigen-positive residents, none lived within the same household as a tongue-positive pig. However, 4/6 (66%) lived within 100 meters of a house where a tongue-positive pig was raised (Figure 2). The odds of being coproantigen-positive were over 9-fold greater for people who lived within 100 meters of a tongue-positive pig (OR 9.4; 95% CI 1.2–71.8).

**Table 3:** Relationship between household characteristics and laboratory results for *Taenia solium* infection in humans, Rica Playa, Tumbes, Peru.

	EITB LLGP		EITB r33		ELISA coproantigen	
Variable	No. positive, (%)	p*	No. positive, (%)	p*	No. positive, (%)	p*
Sex						
Male	75 (39.1)	0.40	6 (3.1)	0.16	2 (1.0)	0.44
Female	67 (34.7)		13 (6.7)		4 (2.0)	
No. residents per household						
1–5 (1^st^ tertile)	77 (41.4)	0.06	7 (3.8)	0.49	3 (1.6)	0.29
6–7 (2^nd^ tertile)	38 (38.0)		7 (7.0)		3 (2.9)	
8–10 (3^rd^ tertile)	27 (27.3)		5 (5.1)		0 (0)	
No. rooms per household						
1–4 (1^st^ tertile)	52 (35.9)	0.51	5 (3.5)	0.45	1 (0.7)	0.31
5–6 (2^nd^ tertile)	65 (35.5)		10 (5.5)		5 (2.6)	
7–9 (3^rd^ tertile)	25 (43.9)		4 (7.0)		0 (0)	
No. houses per 100 meters						
1–5 (1^st^ tertile)	46 (31.7)	0.03	5 (3.5)	0.46	2 (1.3)	0.59
6–9 (2^nd^ tertile)	55 (47.0)		8 (6.8)		3 (2.5)	
10–19 (3^rd^ tertile)	41 (33.3)		6 (4.9)		1 (0.8)	
Latrine in household						
Yes	69 (36.9)	1.0	10 (5.4)	0.82	2 (1.0)	0.69
No	73 (36.9)		9 (4.6)		4 (1.9)	
Pigs raised at household						
Yes	126 (38.2)	0.23	18 (5.5)	0.50	5 (1.4)	0.59
No	16 (29.1)		1 (1.8)		1 (1.8)	
No. pigs per household						
1–3 (1^st^ tertile)	37 (31.6)	0.06	8 (6.8)	0.39	2 (1.6)	0.87
4–8 (2^nd^ tertile)	48 (47.1)		3 (2.9)		2 (1.9)	
9–26 (3^rd^ terile)	41 (36.9)		7 (6.3)		1 (0.9)	
EITB LLGP positive pig in home						
Yes	97 (39.4)	0.69	16 (6.5)	0.47	5 (1.9)	1.0
No	17 (36.2)		1 (2.1)		0 (0)	
Pigs not tested	12 (32.4)		1 (2.7)		0 (0)	
Tongue positive pig in home						
Yes	15 (57.7)	0.09	2 (7.7)	0.72	0 (0)	1.0
No	99 (37.1)		15 (5.6)		5 (1.79)	
Unknown	12 (32.4)		1 (2.7)		0 (0)	
Distance to tongue positive pigs						
0–100 meters	40 (52.0)	<0.01	8 (10.4)	0.08	4 (5.0)	0.02
101–500 meters	30 (27.8)		4 (3.7)		0 (0)	
>500 meters	72 (36.0)		7 (3.5)		2 (1.0)	
Tongue positive pig within 100 m radius						
Yes	40 (52.0)	<0.01	8 (10.4)	0.03	4 (5.0)	0.02
No	102 (33.1)		11 (3.6)		2 (0.62)	

* Fisher's exact test, two-sided.

Of the 385 serum samples, 19 (4.9%) were positive for antibodies against *T. solium* taeniasis on EITB rES33; 17/19 (89.5%) were coproantigen-negative suggesting a history of ingestion of cyst-contaminated pork but absence of current taeniasis infection. Of the 366 samples which were negative on EITB rES33, 4 (1.1%) were coproantigen-positive. Inter-test agreement was poor (kappa = 0.14). As with the coproantigen results, there was no statistically significant difference in the distribution of rES33-positive residents across categories of age (Figure 1), sex or most demographic characteristics (Table 2) other than distance to a tongue-positive pig. Of the 19 total rES33 seropositive residents, 8(42%) lived within 100 meters of a house where a tongue-positive pig was raised. The odds of being rES33 antibody-positive were more than 3-fold greater for people who lived within 100 meters of a house where a tongue-positive pig was found (OR 3.4; 95% CI 1.1–10.9).

### Human Cysticercosis

Of the 385 human sera samples, 142 (36.9%) were positive for antibodies against one or more bands on EITB LLGP for cysticercosis. The positive band distribution on EITB LLGP was as follows: 32 (22.5%) with a single band, 106 (74.6%) with 2–3 bands and 4 (2.8%) with 4–7 bands. In all 32 single-band positive samples the positive band corresponded to the 50 Kd glycoprotein (gp50). There was a significant trend for increasing seroprevalence over increasing categories of age (Figure 1) (p<0.01, χ^2^test for trend). Other significant differences in seropositivity with respect to demographic characteristics are shown in Table 2. Of the 19 rES33 positive individuals, 16 (84.2%) were also positive on EITB LLGP. The odds of positive EITB LLGP were 10 times greater for rES33-positive than rES33-negative individuals (OR 10.2, 95% CI 2.9–35.5). Of the 6 coproantigen-positive individuals, 4 (66.7) were also positive on EITB LLGP. However, there was no significant difference in seropositivity on EITB LLGP with respect to coproantigen results (p = 0.2). The association of seropositivity with distance to a tongue-positive pig was present albeit weaker than with the results for taeniasis. The odds of being EITB LLGP antibody-positive for cysticercosis were 2-fold greater for people who lived within 100 meters of a house where a tongue-positive pig was found (OR 2.2; 95% CI 1.2–4.1). Of the 4 participants with reactivity to >4 positive bands on EITB LLGP, 3 (75%) lived within 100 meters of a tongue-positive pig.

### Relationship between Heavily-Infected Cysticercotic Pigs and Human Taeniasis

Figure 2 illustrates the geographic locations of tongue-positive pigs with their corresponding 100-meter rings, pigs with 4+ bands of EITB LLGP with their corresponding 100-meter rings, and the locations of all coproantigen-positive individuals in the community. The proportion of coproantigen-positive individuals differed significantly between residents living within 100-meters of a tongue-positive pig (4/79, 5.1%) and residents living >100 meters from a tongue-positive pig (2/323, 0.6%) (p = 0.02). There was a significant trend of decreasing odds of positivity with increasing distance from the tongue-positive pig (p = 0.04 trend). A similar pattern was also present for rES33. The proportion of rES33-positive individuals differed significantly between residents living within 100-meters of a tongue-positive pig (8/77, 10.4%) and residents living >100 meters from a tongue-positive pig (11/308, 3.6%) (p = 0.03 homogeneity; p = 0.03 decreasing trend in odds). The crude and adjusted prevalence ratios for these distance categories are shown in Table 4.

**Table 4:** Prevalence of positive laboratory results for *Taenia solium* infection in humans by geographic relation to a household containing at least one tongue-positive pig, Rica Playa, Tumbes, Peru.

	Residents living >100 meters from household with tongue-positive pig *(reference group)* n = 363		Residents living ≤100 meters from household with tongue-positive pig n = 91				
Laboratory assay	Prevalence (%)	95% CI (%)	Prevalence (%)	95% CI (%)	Crude PR	Adjusted PR*	95% CI*
EITB LLGP	33.1	27.8–38.4	51.9	40.7–63.2	1.6	1.3	0.9–1.7
EITB rES33	3.6	1.5–5.7	10.4	3.5–17.3	2.9	3.1	1.2–8.3
ELISA coproantigen	0.6	0–1.5	5.1	0.2–9.9	8.5	8.1	1.4–47.0

CI = Confidence interval.

PR = Prevalence ratio.

* Generalized estimating equations (GEE) with household as the clustering variable and using robust sandwich-type standard errors. Best-fitting models for residents living within 100 meters of a tongue-positive pig included the following variables; 1) EITB LLGP; age and number of pigs raised within the house, 2) EITB r33; number of pigs raised within the house only, 3) ELISA coproantigen; age only.

Constructing 100-meter rings around pigs with 4+ bands instead of around tongue-positive pigs captures an additional coproantigen-positive individual (5/6; 83%). However, there was no statistically significant difference between the proportion of coproantigen-positive individuals living within 100-meters of a pig with 4+ bands (3/288, 1.0%) and residents living >100 meters from a pig with 4+ bands (3/114, 2.6%) (p = 0.4). Nor was the proportion of rES33-positive individuals living within 100-meters of a pig with 4+ bands (11/274, 4.0%) significantly different from the proportion of rES33-positive individuals living >100 meters from a pig with 4+ bands (8/111, 7.2%) (p = 0.2). The crude and adjusted prevalence ratios for these distance categories are shown in Table 5.

**Table 5:** Prevalence of positive laboratory results for *Taenia solium* infection in humans by geographic relation to a household containing at least one seropositive pig with more than 4 reactive bands on EITB LLGP, Rica Playa, Tumbes, Peru.

	Residents living >100 meters from household with tongue-positive pig *(reference group)* n = 326		Residents living ≤100 meters from household with tongue-positive pig n = 128				
Laboratory assay	Prevalence (%)	95% CI (%)	Prevalence (%)	95% CI (%)	Crude PR	Adjusted PR*	95% CI*
EITB LLGP	34.7	29.0–40.3	42.3	33.1–51.6	1.2	1.3	1.0–1.6
EITB rES33	4.0	1.7–6.4	7.2	2.4–12.1	1.8	1.9	0.7–5.3
ELISA coproantigen	1.0	0–2.2	2.6	0–5.6	2.6	2.0	0.3–15.6

CI = Confidence interval.

PR = Prevalence ratio.

* Generalized estimating equations (GEE) with household as the clustering variable and using robust sandwich-type standard errors. Best-fitting models for residents living within 100 meters of a seropositive pig with more than 4 reactive bands on EITB LLGP included the following variables; 1) EITB LLGP; age and number of pigs raised within the house, 2) EITB r33; age and number of pigs raised within the house, 3) ELISA coproantigen; number of pigs raised within the house only.

## Discussion

This study provides evidence of a strong geospatial association in endemic villages between pigs with a heavy-burden of *T. solium* cysts and humans with taeniasis. The prevalence of taeniasis was 8 times higher among residents living within 100 meters of a tongue-positive pig compared to residents living outside this range (adjusted PR 8.1; 95% CI 1.4–47.0). This finding suggests that tongue-positive pigs in endemic communities can indicate geospatial foci in which the risk for human taeniasis is increased. Targeted screening or presumptive treatment for taeniasis within these high-risk foci may be an effective and practical control intervention in rural endemic areas.

Other findings in our study support our conclusion. We observed a similar geospatial association between tongue-positive pigs and humans with serum antibodies against *T. solium* taeniasis (adjusted PR 3.1; 95% CI 1.2–8.3). The slightly weaker observed association is likely due to antibody persistence after cleared infection in some rES33 antibody-positive individuals, as any tongue-positive pigs resulting from the cleared infection would likely have been slaughtered prior to our study. We also observed a weak geospatial association between tongue-positive pigs and human serum antibodies against *T. solium* cysticercosis (adjusted PR 1.3, 95% CI 0.9–1.7), suggesting some concentration of human exposure to *T. solium* eggs within the high-risk foci. However, seropositive pigs and people were widely distributed across the village suggesting that egg exposure is pervasive.

Few other studies have examined geospatial patterns of *T. solium* infection. A previous study in Peru demonstrated that seroprevalence and seroincidence of antibodies against *T. solium* cysticercosis in pigs increases as the distance to a house with taeniasis decreases [24]. However, targeted interventions for taeniasis around seropositive pigs would be impractical as the majority of pigs in an endemic area have been exposed. There was no attempt to verify the true infection status of pigs by tongue examination or necroscopy in that study. Another community study in Peru in which pigs were necroscopied found that nearly half of infected pigs with viable cysts were raised in a house where someone had active taeniasis, while 94% were within 500 meters of a household with taeniasis [unpublished, Cysticercosis Working Group in Peru 2012]. A study of a small village in Mexico reported that none of the 4 taenia carriers found by stool microscopy resided in the same house as a tongue-positive pig [25]. However, actual distances were not reported so geospatial relationships cannot be further assessed. Two other studies in Mexico and Tanzania have shown conflicting results regarding the clustering of infected pigs [26], [27]. However, neither of these studies examined geographic association between porcine cysticercosis and human taeniasis, which our results suggest could provide the basis for a targeted intervention.

While we observed a strong geospatial association between taeniasis and tongue-positive pigs, the relationship was not absolute. There were two people with taeniasis in the community who were not associated with a nearby tongue-positive pig. There are several possible explanations. Recently acquired taeniasis may not be associated with visibly infected pigs, as the latent period between exposure to eggs and development of viable cysts is about 2 months [28]. It is also possible that any heavily-infected pigs associated with these cases of taeniasis were sold or slaughtered prior to our intervention. Finally, it is possible that some cases of taeniasis will simply not lead to heavy infection in a pig due to mitigating host or environmental conditions. Regardless of the explanation, a single cross-sectional approach to targeted screening for taeniasis around tongue-positive pigs would have missed these two individuals. This has important implications for control as an adult tapeworm sheds countless infective eggs into the environment over the course of their lifespan. A single case of persistent taeniasis could potentially maintain transmission in a community. While an alternate strategy of screening around pigs with more than 4 reactive bands on EITB LLGP identified one additional case of taeniasis, this strategy appears less efficient due to the increased number of rings generated and the additional people requiring screening.

It is also important to note that we did not identify any cases of taeniasis occurring within the same household as a tongue-positive pig. This suggests that geographically-targeted screening for taeniasis should not be limited to the source household of the infected pig. Unrestrained pigs will roam beyond their immediate home to forage and may be exposed to *T. solium* eggs in other locales. It is also important to note that the results of the coproantigen test do not provide definitive speciation, and some of the taeniasis we detected could be *T. saginata* rather than *T. solium*. However, we employed a more stringent cutoff for positivity using the coproantigen ELISA in order to reduce the likelihood of detection of *T. saginata*. Our experience in Peru (>100,000 samples processed) has demonstrated that *T. solium* taeniasis produces higher optical density readings than *T. saginata* taeniasis.

Although taeniasis/cysticercosis is considered a potentially eradicable disease, there has been relatively little data published on the effectiveness and sustainability of applied interventions. The TSOL 18 vaccine has been shown to be highly effective in protecting pigs from infection in community settings, but a commercial formulation is not yet available and there are lingering questions about villager uptake in a programmatic setting due to the requirement of multiple doses [29]–[31]. Mass human chemotherapy has been attempted in multiple countries using either niclosamide or praziquantel [9]–[11], and in combination with mass treatment of pigs with oxfendazole [12]. With any of these interventions, a longitudinal approach is important in order to preserve control gains, as demonstrated in Peru where combined human and pig mass chemotherapy showed a return to baseline within 18 months [12]. Persistence of underlying conditions for transmission, decreased herd immunity among pigs, and migration of adult tapeworm carriers into treated areas can promote renewed transmission [20]. Interventions addressing underlying risk factors, including improving animal husbandry practices and sanitary infrastructure, should occur in parallel [32].

As *T. solium* is endemic across several continents, a variety of alternative approaches will likely be required to implement regionally-appropriate control and elimination programs. Studies reporting the effectiveness, practicality and acceptability of candidate control interventions in a variety of settings are urgently needed. The Cysticercosis Working Group in Peru is currently conducting a pilot evaluation of targeting screening for taeniasis around tongue-positive pigs in northern Peru, with initial results expected within a year.

### Limitations

Our study was conducted in a small rural village in northern Peru, a region in which *T. solium* is known to be highly endemic. Our results may not be generalizable to other endemic regions in which the underlying composition of risk factors may not be the same. We chose to analyze a 100-meter radius for targeted screening for taeniasis as this is a practical and easily-replicated distance for implementation purposes. However, housing density, agricultural practices, sanitation, topography and climatic factors may all influence geospatial associations between infected pigs and humans with taeniasis. The factors which promote *T. solium* endemic stability are not fully understood, and it is possible that the foci-centered intervention we propose may not be effective enough to counteract these forces. For example, decreasing pig herd immunity due to reduced exposure to *T. solium* eggs could potentially promote a higher rate of successful infection and ongoing transmission. Finally, this is a small study and analysis is therefore limited to a low number of tongue-positive pigs and taeniasis cases. Our results and conclusions should be validated in a larger endemic population and in areas with higher prevalence of taeniasis.
